# Awareness of postpartum depression among midwives and pregnant women in Arkhangelsk, Arctic Russia

**DOI:** 10.1080/16549716.2024.2354008

**Published:** 2024-06-03

**Authors:** Elena Nechaeva, Olga Kharkova, Vitaly Postoev, Andrej M. Grjibovski, Elisabeth Darj, Jon Øyvind Odland

**Affiliations:** a Department of Public Health and Nursing, Norwegian University of Science and Technology, Trondheim, Norway; b Department of Pedagogy and Psychology, Northern State Medical University, Arkhangelsk, Russia; c Department of Public Health, Health Care and Social Work, Northern State Medical University, Arkhangelsk, Russia; d Department of Health Policy and Management, Al-Farabi Kazakh National University, Almaty, Kazakhstan; e Department of Epidemiology and Modern Vaccination Technologies, Sechenov First Moscow State Medical University (Sechenov University), Moscow, Russia; f Central Scientific Research Laboratory, Northern State Medical University, Arkhangelsk, Russia; g Department of Biology, Ecology and Biotechnology, Northern (Arctic) Federal University, Arkhangelsk, Russia; h Department of Women’s and Children’s Health, Uppsala University, Uppsala, Sweden; i Department of General Hygiene, I.M. Sechenov First Moscow State Medical University (Sechenov University), Moscow, Russia

**Keywords:** Postnatal depression, maternal health, perceptions, pregnant woman, midwife

## Abstract

**Background:**

Postpartum depression (PPD) affects approximately 17% of the women worldwide with nearly half of all cases going undetected. More research on maternal mental health, particularly among healthcare professionals and pregnant mothers, could help identify PPD risks and reduce its prevalence.

**Objective:**

Given that awareness of PPD is a crucial preventive factor, we studied PPD awareness among midwives and pregnant women in Arkhangelsk, Arctic Russia.

**Methods:**

A qualitative study was conducted using in-depth semi-structured interviews. Midwives and pregnant women were recruited from the women’s clinic of the Arkhangelsk municipal polyclinic. Seven midwives and 12 pregnant mothers were interviewed.

**Results:**

Midwives described limited time for psychological counselling of pregnant women; they reported that their primary focus was on the physiological well-being of women. Pregnant women have expressed a desire for their families to share responsibilities. The participants considered PPD as a mix of psychological and physiological symptoms, and they also highlighted a discrepancy between the expectations of pregnant women and the reality of motherhood. The present study underscored the limited understanding of PPD identification.

**Conclusions:**

The findings suggest that there is a need for increased awareness among midwives and pregnant women regarding PPD. Prevention programs targeting PPD with a specific emphasis on enhancing maternal mental health knowledge are warranted.

## Paper context

- **Main findings:** Pregnant women and midwives in an Arctic Russian setting have low awareness of postpartum depression.
- **Added knowledge:** Improved awareness among pregnant women and midwives about the mental health of women after childbirth, educating pregnant women about symptoms of postpartum depression, encourage them to express their needs and collaboration with family supporters may help to reduce postpartum depression burden.
- **Global health impact for policy and action:** Updated campaigns and prevention programs with the focus on increasing the knowledge on mental health among pregnant women and health personnel may be effective support for Primary health care.

## Background

Postpartum depression (PPD) is a non-psychotic disorder many women experience after birth. It can have significant physiological, social and psychological consequences for both the affected women and their families [1]. The first thousand days of a child’s life, from conception until the second birthday, have a significant impact on their health and development [2]. Therefore, maternal mental health is essential for the child’s well-being during this critical period [3], which may be accompanied by maternity blues, PPD and postpartum psychosis [4].

Maternity blues, a temporary shift in mood, may occur within the first 10 days after giving birth. Symptoms may include tearfulness, anxiety, mood swings, irritability, frustration and exhaustion [5]. The frequency of maternity blues may be as high as 83% [6]. While most cases do not require treatment, maternity blues can lead to PPD if left untreated [7]. Maternity blues, a common and transient physiological change, does not lead to negative behavioural consequences such as intrusive thoughts, thoughts of hurting a child or self-harming, or child abandonment [8].

Postpartum psychosis is the most severe form of psychological complications that can occur after childbirth. This condition is characterized by a range of symptoms, including paranoia, hallucinations, confusion, and losing touch with reality. Women who experience postpartum psychosis require immediate medical and psychiatric attention, which often involves hospitalization [9].

PPD is a complex condition that encompasses a range of emotional states that can significantly impact a woman’s mood and behaviour following childbirth. These symptoms can persist for up to two years or even longer. In a study from England, women experienced depressive symptoms for up to 11 years after giving birth [10]. PPD can manifest itself in a variety of ways, including feelings of guilt, sadness, hopelessness, low self-esteem, physical and nervous exhaustion, sleep and appetite disturbances, anxiety, lack of interest in caring for the child, and even suicidal thoughts [11,12].

PPD can have a significant impact on both the mother and the child. The effects may include delayed growth, inhibited emotional development and behavioural issues. Furthermore, the newborn’s decline in cognitive function may increase the risk of depressive symptoms later in childhood [13].

PPD has been shown to be associated with a poor response to the baby’s needs, impaired interaction with the infant, reduced breastfeeding and negative parenting practices [14,15]. Marital conflicts may arise with devastating effects on the family [16].

The global prevalence of PPD was estimated to be 17.2% in 2021 [17]. The prevalence of PPD varies across geographic areas; this variation can be attributed to postpartum care traditions and diagnostic criteria. In 2011, the prevalence in underdeveloped countries was 13% [18]. A recent report has suggested that the prevalence of PPD in Russia may be as high as 34.3% [19].

The data on the prevalence of PPD in the Arctic are limited. Studies from Iceland, Greenland and Norway have reported the prevalence of PPD to be 6.5%, 8.6% and 10%, respectively [20–22]. The prevalence of PPD in Arctic Russia remains unknown [23].

PPD can be identified through conscious observation of behaviour and emotions. A study in the USA revealed that only 40% of the women who experience PPD sought professional help [24]. Cultural tradition may be a factor behind not seeking professional assistance [25]. Considering the increasing prevalence of PPD and its profound effects on mothers, children, and families, it is important to prioritize awareness and prevention of this condition [26]. Awareness of PPD may improve recognition of symptoms and the potential to seek help [27]. Studies have shown that more knowledge about postpartum health was associated with fewer postpartum problems [28,29]. For pregnant women, midwives represent an important source of information about PPD [30].

In Russian maternity healthcare service, midwives work at women’s clinics where they provide outpatient assistance to gynecological patients, pregnant and postpartum women and in maternity hospitals, where they assist childbirth in inpatient settings. During pregnancy, a woman is attended by a medical team consisting of an obstetrician-gynecologist and a midwife in the women’s clinic; during childbirth, a different team, including an obstetrician and a midwife, assists the same woman.

Health education is included in the list of professional responsibilities of a midwife [31]. Through prenatal classes on newborn care and breastfeeding, midwives have a unique position between the woman and the obstetrician playing the central role in preventive activities [32]. In Arctic Russia, the role of midwives is particularly important as they are the only medical staff pregnant women can have contact with in remote areas. Thus, identifying and describing the awareness of PPD among both pregnant women and midwives is important for developing public awareness campaigns for its prevention and timely recognition.

Awareness in this study is closely related to knowledge. There are two concepts of awareness: general awareness and personalized awareness [33]. General awareness is at the lower end of the knowledge continuum and represents the presence or absence of general information. Personalized awareness represents self-perceptions of various health-related conditions [34]. This study aims to explore the general awareness of PPD among both midwives and pregnant women in the Arctic Russian town of Arkhangelsk with the further purpose of creating a preliminary model of a postpartum depression prevention program based on empirical data.

## Methods

### Study setting and study design

The study was conducted at the women’s clinic of Arkhangelsk municipal polyclinic. The population of the Arkhangelsk region was 974,6 thousand in 2022 including 7774 newborn children [35].

This is a phenomenological theory-based qualitative study [36] with individual in-depth semi-structured interviews with open-ended questions [37]. Phenomenology provides researchers with a theoretical guidance for understanding a phenomenon at the level of subjective reality [38]. A pre-assigned interview guide with open-ended questions was deployed to explore knowledge regarding PPD, its symptoms and treatment [39]. Individual semi-structured interviews are an effective method of collecting high-quality, open data to explore the thoughts, feelings, and beliefs of participants on a particular theme [40]. Literature research and studies of the guidelines were performed to search for relevant information and similar studies [41–44]. Suggested questions were expanded and refined in discussions with the research team, which consisted of a childbirth educator and movement therapist, a psychologist and qualitative researcher, two medical doctors, specialized in obstetrics and gynecology with both international research experience and two epidemiologists.

The study was approved by the Institutional Review Board Regional Committee for Medical and Health Research Ethics of Central Norway (REK 134185, 2019) and the Local Ethical Committee of the Northern State Medical University, Arkhangelsk, Russia (Protocol 05/11–19).

### Participants and data collection

#### Midwives (MW)

In 2022, when the interviews were conducted, there were 9 midwives (MW) working in the women’s clinic. One MW oversaw preventive health activities and conducted classes at the antenatal school, one MW worked as the chief midwife and performed administrative functions. Seven MW in the clinic worked in obstetrics-gynecological appointments and attended to gynecological patients, pregnant and postpartum women. The inclusion criteria for MW included residing in Arkhangelsk and working at the Arkhangelsk municipal polyclinic. No specific exclusion criteria were applied. None had a close relationship to the first author (E.N.) who performed all the interviews. E.N. is a childbirth educator with 11-years’ experience who is well acquainted with the studied issue.

Participating MW were recruited via face-to-face distribution of the project information by E.N. during working hours. Having received the information, the MW had a week to consider participation. Interviews with MW were conducted by phone with audio recording at a pre-approved time, consistent with the schedule of midwives. 9 MW gave oral consent and 7 were interviewed before data saturation was achieved (Table 1).

**Table 1.** Midwives’ characteristics.

Midwife	Age, years	Working experience, years	Number of children	Marital status
Midwife 1	Mean = 45 Range = 35–56	20	2	married
Midwife 2		5	3	married
Midwife 3		38	2	married
Midwife 4		15	1	single
Midwife 5		7	2	married
Midwife 6		37	2	married
Midwife 7		36	1	married

#### Pregnant women (PW)

Semi-structured interviews were conducted with pregnant women (PW) attending maternity antenatal care at the polyclinic. PW were selected through purposive sampling [45] based on specific inclusion criteria. We included both primiparous and multiparous women between 28–38 weeks of gestation with an uncomplicated pregnancy, residing in Arkhangelsk and over 18 years of age. Working mothers in Russia are entitled to 140 days of maternity leave. Maternity leave begins 70 days before the estimated delivery date corresponding to 28–30 weeks of pregnancy. This is the period of pregnancy when a woman attends women's clinics, including prenatal schools. Therefore, the women in our study were on maternity leave. PW were recruited through the social network of the antenatal school. PW were contacted via text message with the project information and had a week to consider participation. Twenty-seven PW agreed to take part in the study, and they received a link to an online meeting on Zoom at a prearranged time, eight were unable to attend. The 19 participating PW were informed about the study and gave oral consent. Saturation of the material was achieved when the sample included 12 PW (Table 2).

**Table 2.** Pregnant women’s characteristics.

Pregnant woman	Age, years	Age of gestation, weeks	Pregnancy N/delivery N	Marital status	Job position
Pregnant woman 1	28	33	1/0	married	IT
Pregnant woman 2	41	34	2/0	single	Designer
Pregnant woman 3	27	29	1/0	married	Housewife
Pregnant woman 4	25	30	1/0	married	Teacher
Pregnant woman 5	29	33	2/1	married	Housewife
Pregnant woman 6	35	32	2/1	married	Housewife
Pregnant woman 7	33	30	1/0	single	Manager
Pregnant woman 8	26	33	1/0	married	Teacher
Pregnant woman 9	25	31	1/0	married	IT
Pregnant woman 10	28	30	1/0	married	N/A
Pregnant woman 11	25	29	1/0	married	Manager
Pregnant woman 12	33	35	3/2	married	Housewife

The interviews were conducted in Russian between November 2021 and May 2022, each session lasting between 30 and 60 minutes with a median duration of 50 minutes. The interviews were audio-recorded and transcribed verbatim. The transcripts of the interviews were anonymized by removing any identifying data from the texts. The collection of information occurred until saturation of the material was achieved, meaning that no new information was being added to the study [46]. To accommodate the ongoing pandemic situation, the interviews were conducted online [47,48].

### Analysis

The analysis of the data was conducted using Malterud’s systematic text condensation [49] and Georgie’s psychological phenomenological analysis [50]. The phenomenological approach enabled us to search for the essence of the phenomenon, PPD, through free creative variation. To gain a general understanding, the transcripts were read through several times to identify preliminary themes related to the participants’ awareness of PPD. Each transcript was analyzed independently by the interviewer and two co-authors, ensuring analytical triangulation [51]. The midwives’ transcripts were separately analyzed from those of pregnant women. An interdisciplinary team developed the depth, range of data, and validity of the analysis, and this collaboration continued until the team reached consensus.

The preliminary themes were called unpreparedness for motherhood, idealistic image of motherhood, psychological condition after childbirth, needs of women after childbirth, symptoms of PPD, its onset and prevalence, psychological causes of PPD, expectations and fears.

The next step was to identify meaning units, fragments containing the information on the research question and related to preliminary themes. Then, we coded, which involved identifying, classifying, and sorting meaning units. After meaning units’ condensation and coding, the results were summarized and converted into overall categories, which were divided into subcategories. To illustrate our findings, we have included quotes from MW and PW (Table 3).

**Table 3.** An example of the analytical process of midwives’ and pregnant women’s responses.

An example of the analytical process of midwives’ responses				
Preliminary themes were extracted on a review of the entire data material.	Extraction of meaning units and summarized into codes.		From coding process to overall categories	Categories were divided into sub- categories
	Quotations	Code		
Unpreparedness for motherhood	*“I think that women don’t always know what will happen after childbirth, so it’s important to talk to them about the difficulties they may face”*	Reality after childbirth	Perceived psycho-physiological condition after childbirth	Reality does not match their expectations
Idealistic image of motherhood	*«Women expect everything to be like in beautiful pictures on the Internet, but in reality it may not be the same”*			Before childbirth they have unrealistic expectations
An example of the analytical process of pregnant women’s responses				
Psychological condition after childbirth	*“Feelings of fear before childbirth can lead to tense behaviour during labour and possibly lead to unpleasant consequences for the baby”*	Birth experience	Lack of professional, family and financial needs	Lack of support

## Results

After getting a first impression of preliminary themes, the text was developed into three categories and 10 subcategories related to the themes and illustrated by quotes (Table 4). For protection of participants’ identity, quotes are presented with random numbers, which are not linked to Tables 1 and 2 with participant characteristics.

**Table 4.** Overview of categories and subcategories emerging from in-depth semi-structured interviews with midwives and pregnant women.

Categories	Subcategories
Seen as a mix of psychological and physiological symptoms	Feelings and symptoms of apathy, fear, guilt Bodily and hormonal changes Conflicts between expectations and reality Impaired mother and child relationships
Need for professional and family support	Limited time for psychological counselling Restricted understanding among family members Financial and housing worries
A medical diagnosis or just temporary fatigue?	High prevalence of symptoms Uncertainty about labelling Denial of the problem

Analyses of the MW and PW responses showed similarities in subcategories and categories; this allows us to jointly present and discuss the results of both groups of participants.

### Seen as a mix of psychological and physiological symptoms

The analysis showed that both participating MW and PW discussed a mix of psychological and physiological symptoms after childbirth. These were expressed in the form of perceived feelings, unrealistic expectations after childbirth, and possible negative attitudes towards newborns.

#### Feelings of apathy

PPD was described in various ways: *‘This is a state of a woman’s body, which is expressed in apathy, unwillingness to do anything’ (R2 MW), ‘Well, I don’t know, maybe it’s a loss of joy in life’ (R5 MW)*. Some PW indicated apathy: ‘*I think that it is the lack of desire to do anything. There is no desire, joy and enthusiasm’ (R7 PW)*.

Some talked about the lack of physical strength: *‘This is a state when it is generally difficult for you to even get out of bed, and to do something around the house’ (R11 PW)*. Most MW considered PPD to be a sign of fatigue: ‘*I think, in general postpartum depression is the fatigue and exhaustion of physical strength after childbirth*’ *(R3 MW).*

#### Fear

Some PW were afraid and worried that they would no longer have a normal life: *‘Because life will no longer be the same as before, with the advent of a child, it changes dramatically. And, probably, this restructuring is the reason that a woman falls into depression’ (R10 PW)*. MW also identified fear as a cause of PPD: *‘A woman is afraid to admit that she is weak’ (R3 MW), ‘Women are afraid that this may turn out to be a serious mental illness that their relatives will not understand’ (R1 PW).*

#### Guilt and shame

Due to the wide dissemination of the information about the negative impact of the use of medications and childbirth by caesarean section, a woman may develop a sense of guilt towards the child: *‘Women worry if they had to have an emergency caesarean section and because of this, a feeling of guilt develops in relation to the baby’ (R12 PW), ‘If the birth was difficult, it leaves a strong imprint’ (R2 PW)*. Many PW and MW have reported high expectations during pregnancy, which can lead to feelings of guilt that women cannot cope after childbirth. In addition, their families may not understand such a phenomenon. Consequently, they talked about feeling guilty towards the child: *‘Women in this state cry, get annoyed with the child, and this creates a feeling of guilt’ (R3 MW)*. The stigma associated with mental illness and the problem of PPD contributed to some participants saying that a woman may experience shame. PW have spoken about the difficulty of opening up and sharing a depression, such as in these comments: ‘*A person with depression is not always ready to share, discuss this problem and ask for help*’ *(R3 PW)* and ‘*I wouldn’t tell the doctor at the appointment*’ *(R8 PW)*. Moreover, some PW deny the possibility of psychological ailments after the appearance of the desired child: ‘*I believe that it cannot be that you are expecting a baby, it appears, how can you experience sadness? I can’t even imagine that I wouldn’t want to come to a child*’ *(R5 PW)*. The MW felt that women were ‘*afraid of judgment and misunderstanding from relatives*’ *(R6 MW)* at the thought of recognizing PPD: *‘A woman is afraid of condemnation from her relatives, of getting a stigma that she could not cope with the usual postpartum difficulties’ (R1 MW)*. According to MW, seeking help from a psychologist for many women can become a label for a bad mother, ‘*a woman is afraid of being accused of not coping with the usual postpartum difficulties*’ *(R7 MW).*

#### Bodily- and hormonal changes

Participants described both physiological and hormonal changes in the female body after childbirth which may lead to problems with exhaustion and the risk of PPD.

I think that hormonal changes in the body and severe fatigue are involved in postpartum depression’ (R9 PW). One MW said: ‘After childbirth, oxytocin affects the woman, so it supports the woman after childbirth, and when the uterus stops contracting, the depression can begin. (R4 MW)

#### Conflicts between expectations and reality

The participants mentioned several factors that would increase the likelihood of experiencing PPD, such as high expectations, euphoria, and impaired mother–child relationship.

The PW talked about the difference between expectations during pregnancy and the reality of becoming a parent: *‘I think that the expectations were not the same. That is, when you expect that everything will be easy and you will immediately return to your normal life, but in reality, it turns out not to be so’ (R2 PW)*. MW believed that some women are not ready for their new role as a mother, and it decreases a woman’s mental strength and confidence: *‘It is a role change that a woman takes on and for which she is often not ready’ (R4 MW). ‘I think that women should know how things really happen. I think it’s very important to tell women the truth, to know how everything will be in advance’ (R6 MW)*.

Most women during pregnancy are in a euphoric state when you expect that birth brings you only joy, new impressions, but … reality is not expected (R5 PW), ‘You expect everything to be like in beautiful pictures on the Internet, but in reality it may not match’. (R6 PW)

#### Impaired mother and child relationships

PW indicated that lack of interest in the child and the appearance of negative feelings towards him as a symptom of depression: ‘ *… when the mother does not pay attention to the child at all, or even when the child screams, the mother may hit him’ (R10 PW), ‘[If]A woman develops indifference towards the child, she may not approach him’ (R3 PW)*. A MW described obsessive thoughts about harming the baby as a symptom of postpartum depression: ‘*One of the patients complained to me that she was in such a serious condition that the child annoyed her so much that she wanted to throw him off the balcony’ (R5 MW).*

### Need for professional and family support

The participants discussed the lack of various types of support, which made the situation in the postpartum period difficult. The low level of family support was of particular concern to the participants. They thought that lack of attention from family members could cause PPD. Another problem was the lack of support from the medical personnel, as well as the lack of financial support and adequate living conditions. They also discussed different ways in which PPD could be overcome, by seeking help.

#### Limited time for psychological counselling

Many participants indicated the need for professional support before childbirth. PW hoped that MW would be able to provide them with information about what to expect after childbirth: *‘I believe that it is useful to give information about possible unpleasant situations, physiological changes and emotional background after childbirth before birth’ (R1 PW)*, *‘When the feeling of uncertainty decreases, this always has a beneficial effect, the feeling of anxiety goes away*’ *(R10 PW).*

MW pointed out the limitations of their work schedule at the antenatal clinic: *‘We do not have the opportunity to pay attention to the psychological state of the woman during the appointment, we only do paperwork. Even if the midwife finds signs of depression, she cannot intervene during the appointment’ (R2 MW)*. In turn, PW were aware of the lack of time for medical personnel: *‘I believe that there is very little time at the checkup and there will be no time to talk about any psychological problems*’ *(R2 PW).*

#### Restricted understanding among family members

Many women spoke about the lack of family support: *‘When there is no support from a person from whom you expect it’ (R7 PW)*. They hoped that the husbands would be able to take on some of their household chores: *‘It depends a lot on the husband so he … does not dump all the responsibilities on the woman’ (R9 PW)*. MW believe that PPD can be aggravated due to the lack of support from relatives: ‘*Families are different. After childbirth, a woman needs contact with her family, husband and parents. But there are families where they will say – why are you suffering, everything is fine! It* [the same thing] *was also with me and you will pass’ (R6 MW).*

#### Financial and housing worries

Women were concerned about financial difficulties associated with the birth of the child: *‘I believe that you can give birth to a child only when you have husband, money and housing. Husband, money and housing are the three pillars of motherhood, failure to comply with which can lead to serious problems’ (R5 PW)*. Some PW feared that their careers would be threatened: *‘If you have to continue your work, which brings a good income, then of course this can cause difficulties, fatigue and misunderstanding from the relatives’ (R2 PW)*. MW said that financial and housing conditions have little effect, since they believed that after becoming pregnant, a woman is already ready for these kinds of changes: *‘I think that people are preparing for the birth of a child, so financial and housing conditions are in the last place’ (R7 MW).*

Many PW hoped for an understanding from their relatives: *‘I think you can tell the closest person if you have a trusting relationship with him’ (R11 PW)*. Some PW were deeply convinced of the value of support from their relatives: *‘Only care from relatives can help’ (R9 PW). ‘I believe that first, I would share with my husband. He can suggest, he can support, he is the main support for me’ (R10 PW)*. In addition, PW often mentioned the possibility of getting support from friends: *‘I think you can share with a friend, especially with one who already has children, she can give advice and help find a specialist’ (R7 PW)*. MW recommended contacting their relatives: *‘I would advise postpartum woman to contact relatives. They should definitely help. This is a husband, mother, mother-in-law’ (R2 MW), ‘I always tell women: You have helpers, count on them, let them always help you’ (R4 MW)*.

Women may try to find an opportunity to contact a specialist: *‘A woman can speak with midwife or doctor at the appointment because you can discuss your health with them’ (R8 PW)*. Some said that they intend to seek help and follow the recommendations of the medical staff after childbirth: *‘I would immediately tell them at the first appointment how I sleep, how I breastfeed, how I eat, I would discuss everything at the first appointment’ (R2 PW)*. Moreover, some PW had high hopes for the help of MW after childbirth: *‘A midwife is probably the first person to whom I would tell about my state, she will tell you where to go next. It’s hard to get in touch with a psychologist. And at the first appointment after childbirth, the midwife will immediately advise where to go’ (R4 PW)*. Others did not know how to get help: *‘I would contact a midwife, but I don’t know when and how?’ (R11 PW)*.

### A medical diagnosis or just temporary fatigue?

There were misunderstandings regarding the prevalence of PPD and timing of this ailment.

#### High prevalence of symptoms

Overall, both MW and PW believed that the global prevalence of depression in the postpartum period was high and that PPD occurs in more than 50% in the postpartum period: *‘In my opinion, 80% experience something similar in one way or another*’ *(R5 MW)*. One woman responded that PPD occurs in almost everyone: ‘*It seems to me that almost everyone has depression after childbirth, only in a different form. Some have light depression, some have harder. Well, probably almost everyone has’ (R2 PW).*

#### Uncertainty about labelling

Participants mentioned that depression can begin immediately after childbirth or in the first weeks, ‘*Postpartum depression can begin immediately upon discharge from the hospital, since you have to do everything by yourself at home*’ *(R1 MW)*, ‘*Probably, postpartum depression begins from the first week of a baby’s life, when you don’t know what to take on, because life changes dramatically*’ *(R11 PW)*. Women had difficulties in identifying depression: ‘*Very often I observed this with my friends – there is no mood, they don’t want to do anything, just cry. In fact, it is difficult to understand – is the person just tired or is it a real depression? So, my friend sobbed for the first two weeks after giving birth*’ *(R8 PW).*

#### Denial of the problem

Some PW were convinced that PPD would not affect them: ‘*I don’t even know and I’m not afraid of depression after childbirth*’ *(R12 PW)*. In addition, some women said that there is no need to seek help, but simply recommended resigning themselves to their situation as a temporary complication after childbirth: *‘It seems to me that gradually a woman can cope on her own, living it all together with a child, everything will pass’ (R1 PW)*. Although the MW could admit that they had encountered women with signs of PPD in their practice, they could not determine whether the problem was significant and merited further intervention. For example, one MW reported: ‘*Honestly, I don’t understand the problem of postpartum depression. What’s this? Is it depression? Or… maybe stress, I’m not sure… maybe it’s just temporary fatigue*’ *(R1 MW)*. Furthermore, some MW have argued that the phenomenon of PPD is overestimated and represents the usual fatigue of a woman in the postpartum period*: ‘I think that now postpartum depression is given too much importance, exaggerated. What they talk about and write about is just fatigue’ (R3 MW).*

## Discussion

Our findings suggest that MW and PW share a similar but somewhat unclear understanding of PPD. The participants viewed PPD as a natural condition that affects most women, similar to a study conducted in Australia [52]. However, there was a confusion between PPD and the more common maternity blues, which affects 50–80% of women after childbirth [53,54]. This confusion may be due to the belief that PPD is an inevitable consequence of childbirth caused by stress, and, therefore, therapy is not needed. As in Beck’s study [55], the participants in our study believed that PPD begins immediately after the childbirth, or after discharge from the maternity hospital, which further confirms the confusion between maternity blues and PPD. Interestingly, the participants did not mention the term ‘maternity blues’ during the interviews, indicating a lack of ability to distinguish between normal emotional and physiological adjustments associated with motherhood and PPD. Several participants expressed their dissatisfaction with the lack of support from medical personnel. MW reported that they primarily focus on the physiological state of the women. This observation is consistent with the perception that people tend to prioritize more critical tasks in a complex environment [56]. The perceptions of the midwives’ role in a potential therapy of PPD was limited, due to their recognition of high workload and focus on physical health, which is in line with a study from Iceland [57]. MW often felt powerless as they were unable to assist patients with PPD due to personal or systemic shortcomings. This highlights the need to raise awareness among MW echoing findings from a study in Brazil [58].

The participants identified several symptoms that met the diagnostic criteria for PPD, including depressed mood, loss of interest in daily activities, sleep disturbance, and fatigue as previously described by Sit and Wisner [59]. Interestingly, both PW and MW were more likely to mention external manifestations and changes in emotional state, such as sadness, apathy, self-doubt, shame, lack of joy, and unwillingness to take care of oneself. Furthermore, impairment of the mother’s attitude towards the child, such as ignoring, fear, irritation, anger, and guilt, was a common symptom of PPD. Similar findings were obtained by Milgrom et al. [60].

This study revealed that the participants primarily attributed PPD to psychological and social factors, such as challenging births, unrealistic expectations, and feeling unprepared for motherhood. However, some participants acknowledged the physiological changes that occur during the postpartum period. Reproductive hormones are known to modulate behavioural, emotional, and cognitive responses. Therefore, rapid fluctuations in hormone concentrations during pregnancy and labour are believed to create a susceptible environment for postpartum disorders [61].

Some participants experienced a loss of maternal integrity, leading them to feel ashamed of admitting their symptoms of PPD and increasing their fear of revealing their condition. The symptoms associated with PPD may cause negative emotions such as guilt and low self-esteem, which can affect their quality of life. These results are consistent with previous studies [62,63].

One of the most common comments from the participants was their desire to be seen as a *‘good mother’* [64] who can handle the demands of the motherhood. This desire often leads to comparisons with other mothers and a fear of not being able to cope, which can reinforce the sense of failure. Furthermore, the feeling of shame may arise from misconceptions about PPD due to poor social awareness; which contributes to stigma and may make women less likely to speak about their depression and seek help [65]. It is crucial to increase awareness and understanding of PPD to reduce the stigma and encourage women to seek the help they need.

Our study confirms the significant role of the economic status [66] in the development of PPD. Additionally, we have found that anxiety related to deteriorating housing conditions can also trigger PPD symptoms. These findings highlight the importance of adequate preparation for childbirth, including financial planning and ensuring stable living conditions. As in other studies [67,68], PW in Arkhangelsk often turn to their relatives and friends for support rather than seek help from psychologists. Having an effective social support system has been shown to reduce the psychological burden and increase awareness of PDD and promoting recovery [69].

Family members play a crucial role in the recovery process by providing physical, emotional, and financial support. PW in our study expressed a desire for their families to share responsibilities, such as housework, especially when PPD symptoms arise. This would allow women to focus on prevention and treatment of PPD. All PW hoped for understanding and support from their families, particularly from their husbands.

This study was conducted in Russia, a country with strong family ties, where traditional culture can contribute to PPD [70]. In the contemporary Arctic family, patriarchal views on the role of women and their position in the family are prevalent [71]. Men are considered to be the main breadwinners, while women are expected to prioritize family responsibilities and hide their own needs [72]. Considering that PPD is related to the intra-family situation, understanding and support from husbands is particularly important. The closer the family relationship, the better equipped couples are to cope with the challenges posed by postpartum challenges [73]. Thus, family-oriented preventive measures are crucial in preventing and treating PPD. We revealed that some pregnant women would like to discuss their psychological issues with their midwife and obstetrician at their first postpartum appointment. However, our participants reported being unaware of where to seek help, which was also noted in an Australian study [74]. This lack of knowledge may be attributed to the lack of understanding regarding the physiological nature of PPD, which highlights the absence of a clear treatment algorithm for PPD. Timely detection of PPD can significantly improve a woman’s mental well-being after childbirth [59]. The participants identified the lack of preparedness for motherhood as the primary reason for the gap between expectations and reality. Additionally, there is a significant lack of awareness about depressive disorders among the general population [75,76], which has a profound impact on women’s attitudes and behaviours towards PPD. These findings underscore the importance of promoting awareness about PPD in the society.

The MW emphasized the need for proper antenatal education to prepare women for childbirth and motherhood. PW also expressed the need for antenatal education that covers topics such as psychological and physiological changes that occur after childbirth and how to seek help if needed. Our study indicates that educational programs for PW and their partners are necessary to develop social support during and after pregnancy with the aim to reduce the incidence of PPD and ensure that parents are better prepared for the challenges they may face after the baby is born.

## Strengths and limitations

One of the main strengths of this study is the extensive national and international collaboration with senior researchers with broad expertise in maternal and neonatal health. Their ability to conduct qualitative research across diverse cultures and countries has been valuable in providing a comprehensive understanding of the phenomenon, awareness of PPD, during the investigation. The qualitative study design, through in-depth interviews, has enabled us to gain valuable insights into the participants’ thoughts, behaviours, and perceptions [77].

However, it is important to acknowledge the limitations of this study. The analysis may have been influenced by certain nuances in the translation of texts from Russian into English. It is worth noting, however, that all authors, with exception of two, are fluent in both English and Russian; this has helped to mitigate any potential issues with translation. To separate multi- and primigravida could perhaps have given us more interesting information concerning their awareness of PPD. We did not make this distinction in our analysis of the material as we are aware of the limited number of participants, especially regarding MW. A specific number of participants is not possible to state in advance [78,79]. Normally, the interviews are discontinued when the researcher judges the material to be saturated and no more information is given, in spite of probing questions [46]. We believe we received saturation from the seven interviewed MW.

## Trustworthiness

To ensure the credibility of our study, we initiated a collaborative effort among the authors. This included a systematic analysis of the data, as well as the inclusion of quotes from participants to illustrate the content. To ensure transferability, we provided a detailed description of the context and setting in which the study was conducted, as well as information about the participating respondents. This allows readers to determine if the study is relevant to their own context. Finally, we maintained consistency by conducting the study within a short period of time.

## Conclusions

The results revealed a concerning lack of awareness about PPD among both MW and PW in an Arctic Russian setting. This lack of awareness may lead to physical and psychological discomfort, as well as stigmatization. Therefore, it is crucial to increase public awareness by disseminating accurate information about PPD before childbirth. The results of our study indicate the need to develop evidence-based interventions to raise awareness among MW about the mental health of women after childbirth. MW should take a proactive approach in educating becoming mothers about the symptoms of PPD and reassure them that they are ready to help if symptoms occur, worsen or persist. Additionally, family-centered preventive measures can play a vital role in the recovery of women with PPD. Those caring for the mother and newborn (father, family, extended family) need to be sensitized for symptoms. Women should be encouraged to express their needs for family support during the postpartum recovery process. By taking these steps, we can work towards a society which is better equipped to handle the challenges of PPD and support new mothers in their journey towards recovery.
